# Recent Advances in Antioxidant Polymers: From Sustainable and Natural Monomers to Synthesis and Applications

**DOI:** 10.3390/polym13152465

**Published:** 2021-07-27

**Authors:** Chrysanthos Maraveas, Ilker S. Bayer, Thomas Bartzanas

**Affiliations:** 1 Department of Natural Resources and Agricultural Engineering, Agricultural University of Athens, 11855 Athens, Greece; t.bartzanas@aua.gr; 2 Smart Materials, Istituto Italiano di Tecnologia, 16163 Genova, Italy

**Keywords:** antioxidant, antioxidant polymers, lignin polymers, graft polymers, dopamine, polydopamine, inulin, quercetin, limonene, vitamins

## Abstract

Advances in technology have led to the production of sustainable antioxidants and natural monomers for food packaging and targeted drug delivery applications. Of particular importance is the synthesis of lignin polymers, and graft polymers, dopamine, and polydopamine, inulin, quercetin, limonene, and vitamins, due to their free radical scavenging ability, chemical potency, ideal functional groups for polymerization, abundance in the natural environment, ease of production, and activation of biological mechanisms such as the inhibition of the cellular activation of various signaling pathways, including NF-κB and MAPK. The radical oxygen species are responsible for oxidative damage and increased susceptibility to cancer, cardiovascular, degenerative musculoskeletal, and neurodegenerative conditions and diabetes; such biological mechanisms are inhibited by both synthetic and naturally occurring antioxidants. The orientation of macromolecules in the presence of the plasticizing agent increases the suitability of quercetin in food packaging, while the commercial viability of terpenes in the replacement of existing non-renewable polymers is reinforced by the recyclability of the precursors (thyme, cannabis, and lemon, orange, mandarin) and marginal ecological effect and antioxidant properties. Emerging antioxidant nanoparticle polymers have a broad range of applications in tumor-targeted drug delivery, food fortification, biodegradation of synthetic polymers, and antimicrobial treatment and corrosion inhibition. The aim of the review is to present state-of-the-art polymers with intrinsic antioxidant properties, including synthesis scavenging activity, potential applications, and future directions. This review is distinct from other works given that it integrates different advances in antioxidant polymer synthesis and applications such as inulin, quercetin polymers, their conjugates, antioxidant-graft-polysaccharides, and polymerization vitamins and essential oils. One of the most comprehensive reviews of antioxidant polymers was published by Cirillo and Iemma in 2012. Since then, significant progress has been made in improving the synthesis, techniques, properties, and applications. The review builds upon existing research by presenting new findings that were excluded from previous reviews.

## 1. Introduction

### 1.1. Definition of Antioxidants. Their Importance in Food Preservation

Antioxidants are a class of naturally occurring or synthetic compounds. The naturally occurring antioxidants include Vitamin C and Vitamin E (tocotrienols and tocopherols in general) [1,2,3]. Other classes are phenolic compounds and carotenoids [4]. Synthetic antioxidant molecules include α-lipoic acid, N-acetyl cysteine, melatonin, gallic acid, captopril, taurine, catechin, and quercetin [5]; these compounds are indispensable in the scavenging of free radical species. For example, gallic acid and poly Trolox ester polymers scavenge free radicals in the cytosolic cellular compartment [6]. In contrast, β-carotene, and vitamin E (tocopherol), are most effective against lipid peroxidation.

Wattamwar et al. [6] attribute the formation of reactive oxygen species to cellular processes, including inflammation—a process that results in the activation of endothelial cell macrophages, which in turn trigger the development of Nicotinamide adenine dinucleotide phosphate (NADPH) oxidase complex [6]. The NADPH complex is responsible for the conversion of molecular oxygen into O_2_, a superoxide reactive anion, which is converted into H_2_O_2_ (hydrogen peroxide) following contact with superoxide dismutase [6]. The hydrogen peroxide molecule is a potent reactant, which binds with copper and iron metal ions, releasing hydroxyl radicals. Alternatively, ONOO^−^ (peroxynitrite) is formed if the superoxide reactive anion is formed in a NO-rich environment. The reactive oxygen and nitrogen species formed through a sequence of reactions triggered by NADPH are responsible for cellular oxidative and lipid damage—phenomena that result in the release of oxidative stress markers (4-hydroxy-2-trans-nonenal, 3-nitrotyrosine (3NT), protein carbonyl). The primary function of melatonin, gallic acid, captopril, taurine, catechin, and quercetin, vitamin C, and E analogs is to bind to the markers to prevent structural damage to cellular proteins.

The biological importance of different antioxidants in food preservation and augmentation of body defense mechanisms depends on chemical properties. Pryor et al. [3] evaluated the performance of both natural and synthetic antioxidants in sodium dodecyl sulfate micelle solutions; it was noted that hydrogen bonding on the para and ether O_2_ atoms predicted the reactivity of the antioxidants towards peroxyl radicals. Beyond H-bonding affinity towards reactive oxygen species was predicted by the presence of bulky tert-butyl groups [3]. Even though synthetic and natural antioxidants exhibit similar potency towards ROS, the selection of the chemicals in industrial applications is predicted by regulatory standards. European Parliament and Council Directive No.1333/2008 restricts the use of synthetic antioxidants in food preservation [4], except for 2, 4-dichlorophenoxyacetic acid (2,4-DA), 2-naphthol (2NL), 4-phenyl phenol (OPP), tert-butyl hydroquinone (TBHQ), butylated hydroxytoluene (BHT), and butylated hydroxyanisole (BHA) [4]. The listed synthetic antioxidants have found broad application in the food industry, particularly in preserving fruits and vegetables, due to their low cost, stability, performance, and widespread availability [3,4,5,7].

Vitamin C has been proven to exhibit superior activity in the quenching of reactive oxygen species and free radicals, resulting in the formation of ascorbyl radicals. The latter is a less potent radical compared to ROS based on the potential for oxidative damage [2]. Vitamin E exhibits a similar mechanism of action as Vitamin C. However, in the former case, the main mechanism of action involves protecting biological liquid compartments and the cleavage of the lipid peroxidation chain reactions. Alternatively, tocotrienols and tocopherols, in general, have been proven to contribute to the inactivation of ROS before regeneration by ascorbate [2]. Experimental evidence has also demonstrated the practical benefits of chain-breaking tocopherols in mitigating the auto-oxidation of polyunsaturated fatty acids. This process is responsible for cancer cell growth and atherosclerosis, as well as other life-threatening conditions [3]. Other unique biological functions associated with naturally occurring antioxidants include the modulation of the activity of specific enzymes, including mitogen-activated protein kinase, protein tyrosine kinase (PTK), protein tyrosine phosphatase (PTP), protein phosphatase 2A (PP2A), and protein kinase [2].

The commercial suitability of naturally occurring antioxidants is informed by various considerations beyond regulatory standards (European Parliament and Council Directive No.1333/2008) on food preservation, encompassing antimicrobial and antifungal activities [4], lipid oxidation reactions, and chemical reactions related to anti-oxidative effects [3,4,5]. The synergistic impact of different considerations helps explain why phenolic antioxidants are often preferred in food preservation (see Table 1).

The extraction techniques predict the selection of natural antioxidants for food preservation. Complex extraction techniques such as microwave-assisted extraction (MAE), ultrasound-assisted extraction, pressurized liquid extraction (PLE), high hydrostatic pressure (HHP), and supercritical fluid extraction (SFE) and methanol, ethanol, acetone, and water predict the cost of the extraction process and industrial application [1] (see Table 2).

Natural and synthetic antioxidants are integral to reducing oxidative stress and imbalances in the cell redox reactions associated with reactive oxygen species. The formation of ROS is linked to the suppression of the innate antioxidant defense systems or the overproduction of oxygen species. Both mechanisms are common, considering that oxygen is ubiquitous in the environment, especially in biological processes involving aerobic organisms [2]. The visual illustration suggests that free radical scavenging ability is predicted by chemical structure and active components.

#### Supercritical Carbon Dioxide-Based Techniques

New evidence shows that the development of active packaging films is augmented by supercritical carbon dioxide (SC−CO_2_) impregnation [8]. The observations made by Franco et al. [8] were collaborated by Lukic et al. [9] and Trucilo et al. [10], who documented the development of active food packaging products made of PLA/PCL combined with thymol and/or carvacrol and liposomes. The antioxidant and packaging properties of the active packaging materials were augmented by supercritical carbon dioxide (SC-CO_2_) impregnation [9]. The choice of the supercritical CO_2_ is grounded in its ability to yield good material properties based on its near-zero surface tension, low viscosity, and high diffusion coefficient [10]. The suitable material properties partly explain why SC-CO_2_ has been extensively applied in membrane formation, desorption, micronization, and extraction. However, in contrast to Franco et al. [8], Lukic et al. [9], Trucilo et al. [10], Ozkan et al. [11] and Cejudo et al. [12] have argued that the utility of SC-CO_2_ in active packaging films was dependent on the role of the material (co-solvent, anti-solvent, or swelling agent). On the one hand, SC-CO_2_ can function as a supercritical anti-solvent (this is critical in micronization or co-precipitation processes). On the other hand, it can function as a solvent or co-solvent. Following the appraisal of the two techniques, it was clear that the SC-CO_2_ supercritical anti-solvent process (SAS) is highly appropriate in packaging given it yields products with customized properties such as spherical nanoparticles, and nanostructured filaments with controlled mean size and particle size distribution.

### 1.2. Natural Phenolic Polymers (Lignin and Proanthocyanidins (PAs))

Lignin is a naturally occurring polymer/heterogeneous biomacromolecule that supports the connective tissues in plants [13,14,15]. The rigidity, tensile strength, resistance to chemical degradation, and pressure-resistance of lignin are linked to the phenyl propane created using coniferyl alcohol, p-coumaryl alcohol, and sinapyl alcohol units [16]—precursor-specific structural modifications of lignin translate to unique, different structural modifications. The microscopic-level changes improve the utility of the material in the production of plastics, paints, films, PU-based forms, nutritional supplements, resins and coatings, beverage additives, food additives, adhesive binders, carbon fiber composites, and enhancement of the structural properties of existing materials [17]. The application of lignin-based compounds in dopamine polymerization, synthesis of polydopamine and copolymers, polymerization of inulin, enzyme-catalyzed polyphenol, polymerization, antioxidant quercetin polymers, polyquercetin and quercetin copolymers, and antioxidant properties, and antioxidant terpene polymers is explored under Section 2 and Section 3. 

Proanthocyanidins are a class of natural phenolic compounds derived from natural phenols (flavan-3-ols) and are fundamental polyphenolic components of the human diet, including red wine, cocoa, dark chocolate, orange juices, grapefruit, parsley, black and green tea, onions, olives, broccoli, and apple skin [18,19]. The PAs are critical to the investigation of sustainable and natural monomers, synthesis, and applications of antioxidant polymers considering that they are the second most naturally occurring and abundant plant polyphenols after lignin [18]. In addition, PA compounds possess ideal chemical properties for industrial and biological applications. For example, cinnamon-derived polyphenols have been proven to have superior anti-inflammatory characteristics–a factor that is instrumental in the treatment of inflammation-related conditions, including inflammatory bowel disease, rheumatoid arthritis, asthma, cancer, diabetes, cardiovascular diseases, degenerative musculoskeletal diseases, and neurodegenerative conditions. The need for plant-based alternatives is supported by the limitations of traditional non-steroidal anti-inflammatory drugs (NSAIDs) [20], the manifestation of appropriate antioxidant properties [21,22], and a variety of naturally occurring precursors, including agro-wastes.

### 1.3. Recovery of Antioxidants from Agro-Wastes

The recovery of antioxidants from agro-wastes is a facile and scalable method in the manufacturing of a broad array of lignin-containing compounds for industrial and biological applications. Martinez-Avila et al. [23] noted that the fermentation of fruit peel waste generated high-quality phenolic antioxidants for industrial application. However, the recovery of antioxidants from agro-wastes is impacted by production-related constraints—traditional processes are associated with significant hazards to human health, low yields, and negative ecological effects; this explains why solid-state fermentation and solid-state shear pulverization have become the techniques of choice in the recovery of polyphenols from agro-wastes [23,24]. From a manufacturing perspective, the technical constraints are offset by the cost advantage. The cost of agro-waste is about USD (United stated dollars) 0.05–0.10, which is significantly low compared to synthetic antioxidants, which cost > USD 6 per kg (Table 3) [24]. Future advances in technology might resolve the low yields, hazards to human health, and negative ecological effects associated with toxic products.

In theory, it is feasible to extract antioxidants from nearly all commonly available agricultural wastes. Nonetheless, there is a strong preference for antioxidant-rich precursors such as orange peel, coffee grounds, turmeric shavings, and waste and grape pomace wastes, which exhibit superior performance in the reinforcement of plastic properties.

The antioxidants are incorporated into polyolefins to enhance their material properties, primarily recyclability, weathering resistance, and high-temperature processability [23,24], which vary in line with the chemical properties of the precursors/agro-waste materials. The presence of hydrogen-donating hindered phenols in the agro-waste materials is integral to the capture of alkyl peroxide radicals, a process that impedes the propagation of free radical reactions [24]. The precursor-specific chemical behavior underscores the need to select appropriate natural antioxidant molecules for antimicrobial treatment, biodegradation of polymers, tumor-targeted drug delivery, and food fortification.

### 1.4. Natural Antioxidant Macromolecules

Antioxidant polymers are a broad class of polymers, including polylactones [25], polymer nanoparticles integrated with living polymerization techniques [26], poly(b-malic acid) derivatives [27], lignin graft polymers, and polyphenols, which offer unparalleled capabilities in tumor-targeted drug delivery, food fortification [28], biodegradation of polymers, and antimicrobial treatment [29]. Emerging research evidence indicates that hydrophobic antioxidant polymers are effective corrosion inhibitors in steel structures.

The development of green, biocompatible, and biodegradable antioxidants is critical in polymer processing industries, packaging, health, cosmetics, textiles, and agricultural sectors. The transition to green materials is supported by growing environmental awareness. The need to explore antioxidant molecules is supported by widespread availability in nature, such as lignin and polyphenol adduct polysaccharides. Additionally, certain natural essential oils such as limonene also feature antioxidant properties and can be polymerized into renewable polylimonene while preserving their antioxidant properties. The antioxidant polymers (including polyfurfuryl alcohol and its derivatives) can be directly sourced from agricultural wastes. Polyphenols drawn from a variety of sources, including fruits and vegetables, dopamine, pyrogallol, tannic acid, and green tea catechins, undergo controlled oxidation and bio-inspired polymerization, which is vital in biomedical applications. For example, quercetin (a potent antioxidant) is polymerized to encapsulate cancer treatment medicines. Recently, edible flavors such as vanillin (phenolic aldehyde) have also been polymerized into an antioxidant copolymer known as polyoxalate co-vanillyl alcohol (PVAX) for biomedical applications. Vitamin C and other poly vitamins with potent antioxidant properties can facilitate the synthesis of ADMET, acyclic diene metathesis polymerization. The procedure involves a step-growth polymerization where α, ω-diene monomer is transformed in the presence of a ruthenium catalyst.

In contrast to synthetic antioxidants, natural antioxidants are naturally occurring compounds generated by plants, fungi, bacteria, and mammals, and are environmentally benign and largely biocompatible. Additionally, natural polymers are preferred due to the diminished risk of harmful byproduct formation in the course of their use relative to synthetic antioxidants [30]. Common antioxidant polymers include essential vitamins such as retinoids, ascorbic acid, tocopherols, and polyphenolic compounds epigallocatechin gallate (EGCG), quercetin, curcumin, and resveratrol [31]. The natural antioxidants exhibit unique material properties, including the ability to scavenge reactive oxygen species, targeted delivery, and long-term functionality/reactivity [32]. Such properties are vital agricultural, textiles, cosmetics, health, and packaging industries. Future applications require the development of materials with customized properties for specialized applications.

### 1.5. A Review of Antioxidant Molecule-Incorporating Polymers

The encapsulation of antioxidant molecules in conventional polymers is integral to the regulation of the potential toxicity, lability, solubility, and diffusion, and other antioxidant properties; this is particularly true for natural antioxidant polymers, which achieve high antioxidizing effectiveness at relatively low concentrations [33]. In other cases, the incorporation of antioxidant molecules into polymers has been proven useful in autosynergism and heterosynergism [33]. Lith and Ameer [32] and Sawant et al. [1] reported the successful encapsulation of vitamin C antioxidant polymer in PEO (or poly(ethylene glycol), PEG) micelles. The incorporation of vitamin C into PEG/PEO micelles was beneficial in the treatment of cancer. Preliminary in vivo experiments using mouse models demonstrated selective necrosis of breast cancer cells; the outcomes demonstrate the unique capabilities of antioxidant compounds embedded into polymers [1,32]. However, the potency of the antioxidant polymers was dependent on concentration—higher concentrations were established to be highly effective in overcoming cancer antioxidant defense mechanisms. Wang et al. [26] reported successful incorporation of paclitaxel into polyethylene glycol-b-poly(D, L-lactide) (PEG-PDLLA) micelle for tumor-targeted drug delivery. Preliminary clinical data show that the technique offers promising prospects in the treatment of tumors.

Beyond targeted drug delivery, the encapsulation of antioxidant molecules in conventional polymers was proven beneficial in agricultural and food packaging applications. The incorporation of primary phenolic antioxidants into polyolefins and polyethylene has been proven useful in the protection of the thermo-oxidative stability of polymers used in packing [30]. The stabilization of synthetic polymers exposed to natural elements, including UV radiation, shear forces, and higher temperatures, is integral to their durability post-production. However, there are ecological implications associated with the incorporation of natural phenolic antioxidants into polymers [30]. Some studies have suggested that the incorporation of antioxidant polymers into polymers poses significant environmental and health hazards. For example, the study found traces of plastics (polyethylene) in drinking water [34]. The problem was attributed to the byproducts of phenolic antioxidant additives used in pipeline production [34]. The compounds migrated to waterways via diffusion. The presence of polymers in drinking water elevates the risk of health complications associated with reactive oxygen species [32]. Even though the latter findings point to the negative effects of antioxidant molecules in conventional polymers, there are tangible benefits associated with the embedding of antioxidant species. The positive benefits in the agricultural, textiles, cosmetics, health and packaging industries help to offset the adverse effects.

## 2. Lignin Polymers and Antioxidant Properties

The review of lignin polymers as potential antioxidants for biodegradation of polymers and antimicrobial treatment [29], tumor-targeted drug delivery, food fortification [28], and diabetes treatment [15] is supported by the chemical composition of lignin structures, abundance in the natural environment, and ease of production; conservative estimates suggest that at least 70 million tons of lignin are generated from pulp [35]—of which 2% is converted to value-added products including lignin for biological, pharmaceutical and chemical industry applications [35]. The widespread availability of lignin in plant species predicts cost. The availability of lignin is predicted by the rate of lignin biosynthesis, which is responsible for biotic and abiotic stress management, organ/tissue development, and growth [36]; these processes are mediated by a broad class of enzymes and genes. Higher expression of CCR, CCoAOMT B, C3H, 4CL, and F5H was associated with pronounced tissue growth [37]. Since the genes predict the presence of G, S, and H units [16], the genetic composition of the different plant species strongly predicts the utility of the plant lignin in tumor-targeted drug delivery, food fortification [28], biodegradation of polymers, and antimicrobial treatment [29]. The observations made by Liu et al. [16] are valid, considering that the G, S, and H units which comprise sinapyl alcohol, coniferyl alcohol, and p-coumaryl alcohol, respectively, predict the rate of lignin monomer copolymerization. The function of different genes on plant species is heightened in Table 4.

The focus on selected plant species as precursors for lignin-based polyurethanes, dopamine polymerization, polydopamine and copolymers, polymerization of inulin, enzyme-catalyzed polyphenol polymerization, antioxidant quercetin polymers, polyquercetin and quercetin copolymers, and antioxidant properties, antioxidant terpene polymers, polylimonine synthesis and properties, antioxidant random copolymers, and gallic acid grafted polymers is beyond the scope of this research inquiry. This means that the desired chemical properties of lignin-based compounds are considered in general without a specific emphasis on a particular plant species.

Lignin molecules have vast antioxidant properties due to the presence of certain functional groups such as p-hydroxy acetophenone extracted from oil palm fronds [38]. The antioxidant properties of lignin-derived polymers are predicted by the precursors. Different precursors have different antioxidant properties, which are predicted by the presence of carbohydrates, G + S phenols, Pi-conjugated carbons, ArC (Py-products with primary, secondary and tertiary carbons on the side chains), carboxylic groups, OH groups, and phenols [14]. The number of functional groups that predict chemical behavior considering the chemical structure of lignin is poorly understood—the aliphatic OH and phenol groups increase the probability for functionalization of the compound [10] and biological and pharmacological function. The antioxidant properties and higher binding affinity of the chemical functional groups in lignin enable the material to bind to bile acids in the intestines—a process that facilitates serum control.

The antioxidant properties are also integral to tumor suppression—animal models suggest that lignin reduces the adverse effects associated with different carcinogens, including 3,2-dimethyl-4-ami-biphenyl [15]. The phenolic compounds isolated from the different lignin structures have been proven to inhibit microbial growth through the inhibition of oxygen-mediated reactions, ATP depletion, and interference with the intracellular pH [35]. In other cases, organic functional groups within the lignin structure (carvacrol, thymol, and cinnamaldehyde) trigger bacterial lysis and damage the cell membrane [35]. The lignin compounds extracted from corn Stover have exhibited appropriate antioxidant properties in eliminating free radical initiators in the red blood cells such as AAPH (2,2-azobis (2-amidinopropane)) [35]. The trends and applications of lignin as a natural antioxidant supersede the antimicrobial effects; this means that natural lignin best functions as a free radical scavenger/borrower [35]. The utility of the antioxidant properties transcends pharmaceutical applications—the chemical antioxidant properties offer protective benefits to the skin and eyes; this means that topical formulations can be prepared from lignin extracts for cosmetic applications. Other potential commercial applications can be explored due to the heterogeneity of lignin structures drawn from diverse sources. Moreover, research has demonstrated that it is possible to customize the behavior of lignin in different applications to stimulate the desired biological function [35].

Even though diverse plants are a suitable source of lignin, only selected sources are used for commercial applications due to wide variations in the lignin composition by weight 10–40 wt%, the need to balance resource use and promote green economy [39]. In other cases, the herbaceous weight composition of biomass is lower 15–25% (*w*/*w*) [15]; this explains why most lignin is derived from byproducts during pulp production [15,39]. The production-related requirements have practical implications on the choice of lignin precursors for biological, polymer processing industries, agricultural, cosmetics, textiles, and agricultural applications. This observation is supported by the link between the precursor and chemical properties and production methods [35]. Certain sources are associated with a higher cost of production, low yield extraction, higher energy use, poor solubility, and presence of chemical impurities; this means that the choice of fractionation methods of technical lignin predicts commercial utility [35].

Even though precursor-specific chemical functional groups might be ideal in the development of lignin-based antioxidants for pharmaceutical, medical and packaging applications, other factors have to be taken into account, including the availability of the plant species and lignin content. The *Quercus* plant species have an extremely low lignin content (3.8%); this is in contrast to 54% in *Acacia auricuriformis* [36]. The high concentration of lignin in the latter justifies its use as a preferred source of antioxidants for a broad array of applications. However, there is significant information asymmetry about the performance of different plants with variable concentrations of lignin—most studies focus on selected plant species [14], with proven benefits. The bias towards selected plant species could have practical implications in commercial applications.

### 2.1. Lignin Graft Polymers and Copolymers

The development of lignin graft polymers and copolymers is integral to the sustainability of modern civilizations and human life. However, there are critical constraints in the synthesis of lignin-based high-performance materials [40]. Most lignin-based materials have been used in the development of low-value products such as pesticides, animal feeds, surfactants, binders, dyestuff dispersants, concrete additives, and materials for dust control, which account for about 2% of the total fraction of lignin produced globally [40]; this means that there is a clear mismatch between the rates of lignin production and the development of value-added products. A feasible approach for the synthesis of value-added lignin products is the derivatives that entail the development of lignin graft polymers and copolymers.

Modern plastics are primarily sourced from petroleum byproducts, but there are valid and emerging concerns about the environmental implications, greenhouse emissions, and destruction of marine and terrestrial environments [41,42,43]. The growing environmental concerns have validated the need to develop sustainable methods for reducing the carbon footprint associated with plastics; this has, in turn, triggered the development of bio-based plastics for agricultural and packaging applications [44,45,46]. A fundamental constraint includes the shortage of suitable precursors for bio-based plastics. Chitosan and corn are less abundant, pose a direct threat to commercial agriculture, and commercial viability is challenging [45,47], making it unfeasible to upscale operations. The highlighted challenges demonstrate the need for alternative and complementary sources of sustainable and biodegradable plastics; this informs the development of lignin graft polymers and copolymers for sustainable plastic materials. The focus on lignin in place of other forms of biomass is grounded on the natural abundance in plant and tree species [36,37]. The relative composition varies between 10 and 40 wt% [15,39], depending on the tree species, which is significant considering that about 70 million tons of pulp are generated [35]. The pulping byproducts are rich in lignin; this means that the precursor can be extracted using a scalable and low-cost method. Affordability is integral to the gradual phasing out of synthetic plastics with biodegradable materials in emerging and developed economies.

Lignin graft polymers have been synthesized using a variety of techniques, including blending natural lignin extracted from plants with commercial-off-the-shelf polymers [13] to augment the mechanical and thermal properties of the polymer blends. Copolymer blends have other desirable properties, including superior gene delivery properties, surfactant properties, UV absorber properties, super-plasticizer capabilities, and enhanced elasticity [16]. The realization of these properties is dependent on controlled polymerization and the selection of the backbone structure/polymers and the branch polymers [13,16,48]. The two are connected via covalent bonding.

The properties of the blends are predicted by a broad range of factors, including the polymerization techniques, click reactions, reversible-addition-fragmentation chain-transfer (RAFT) polymerization, and radical-mediated lignin-graft copolymerization [13,16,48]. Other feasible synthetic techniques include single-electron transfer living radical polymerization (SET-LRP), atom transfer radical polymerization (ATRP), macromolecular design via the interchange of xanthates (MADIX), and single-electron transfer-degenerative transfer living radical polymerization (SET-DTLRP) [49]. Even though there are multiple feasible copolymerization techniques, ATRP and RAFT are widely used.

**Figure 1:**
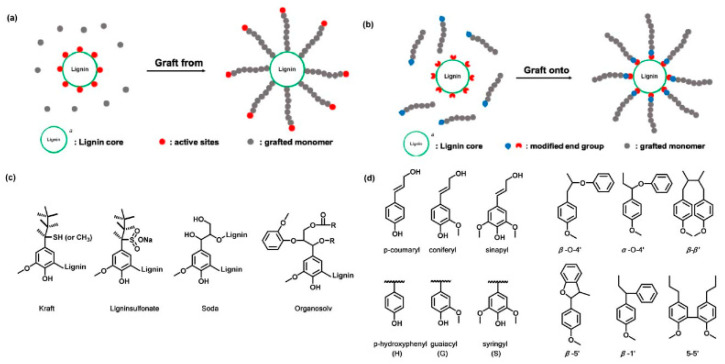
(**a**) An illustration of the graft-from synthesis of lignin copolymers; (**b**) a demonstration of graft-onto methods; (**c**) industrial lignin products; (**d**) sinapyl alcohol, coniferyl alcohol, and p-coumaryl alcohol units [16].

The attainment of the desired properties during polymerization is contingent on graft density, the length of the grafts, and the functional groups on the graft polymers. The most commonly used grafting techniques (Figure 1) include grafting-through, grafting into, and grafting from [16]. The graft-from technique is characterized by the grafting of the polymers from active sites at the backbone polymer (lignin).

**Figure 2:**
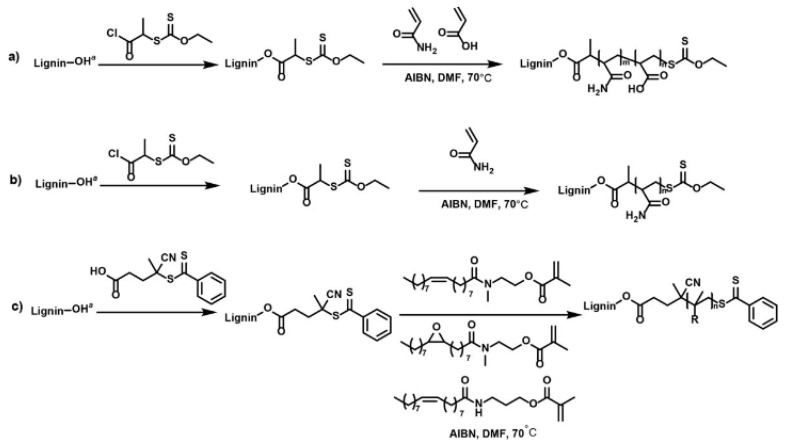
RAFT polymerization demonstrating: (**a**) acrylic acid and acrylamide graft-from polymerization; (**b**) RAFT polymerization of acrylamide; (**c**) soybean oil polymerization using the graft-from technique. The lignin compound is comprised of both phenolic hydroxyl and aliphatic hydroxyl groups [16].

The grafted polymers emerge from the backbone as a result of radical polymerization, ring-open polymerization, RAFT, and atom transfer radical polymerization (ATRP) [16,48] (see Figure 2). Each polymerization technique has its benefits and constraints. For example, ATRP relies on an alkyl halide initiator, which is then radicalized, resulting in the polymerization of monomers [13]; this process is augmented by a transition metal catalyst. In contrast, RAFT polymerization is augmented by radicalization from the addition of a radical-generating species [13,16], while ROP is characterized by the opening of a cyclic molecule in the polymerization process; this results in the formation of a reactive species that undergo a chemical reaction with the next cyclic molecule [13]. The choice of either copolymerization process is predicted by a broad range of factors, including cost, the quality of the desired product. For example, Liu et al. [16] argue that RAFT is an ideal technique for newly made monomers, and ATRP is appropriate for commercially available acrylate monomers. In the former case, the lignin backbone is modified through the incorporation of an ester linkage to create a RAFT agent moiety; this procedure is subsequently followed by the polymerization of the vinyl monomers.

The choice between RAFT and ATRP entails a tradeoff of material properties and the synthesis process. Lai et al. [50] suggest that RAFT is highly suitable for commercial applications relative to ATRP and other synthetic techniques [50] due to the incorporation of acrylic derivatives and organic substances. The bio-applications of RAFT polymerization documented by Boyer et al. [49] corroborate the assessment of Lai et al. [50] of the key benefits associated with RAFT. For instance, RAFT features most of the desirable aspects of traditional free radical polymerization, namely facile reaction conditions, tolerance of most functionalities, site-specific functionality, control of molecular weight distribution and molecular weight, and compatibility with a wide array of monomers. In contrast to traditional living radical polymerization (LRP), RAFT features site-specific functionality [50]. The commercial significance is offset by synthetic requirements.

The RAFT polymerization technique depends on the ability of RAFT agents to transport an ideal leaving group bonded to an S-atom to the reaction site; it is often challenging to attain such reaction specificity. In view of these constraints, the RAFT polymerization has low yields. There are also concerns about the synthesis of toxic compounds, namely Grignard reagents [50]. The inadequacy of RAFT and ATRP techniques demonstrates that new methods are required to meet the need for the synthesis of next-generation lignin graft polymers and copolymers.

### 2.2. Lignin-Based Polyurethanes

**Figure 3:**
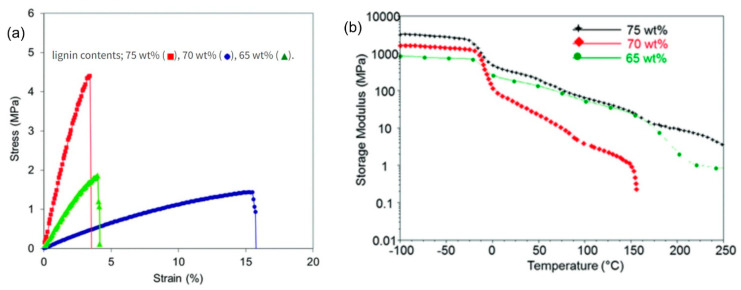
(**a**,**b**) Stress–strain behavior and storage modulus of lignin-based polyurethane thermoplastics [40].

The synthesis of lignin-based thermoplastic polyurethanes has been demonstrated through copolymerization [40]. Lignin precursor is incorporated into the backbone of the polyurethane structure, but most of the products are rarely recyclable or processable due to extensive crosslinking and networking within the polymer structure [7,40,51]; this means that the polydisperse nature of lignin molecules is a critical impediment to the synthesis of thermoplastic polyurethanes. Saito et al. [40] suggested that the challenge could be offset through the incorporation of tie molecules to link the hard segments or crystals. The synthesis of recyclable thermoplastic lignin-based polyurethanes could also be augmented by bridging the soft segment (characterized by a low glass transition temperature T_g_) with the hard segments containing lignin to ensure that the final product features the mechanical rigidity of lignin and the soft rubbery properties of the soft segments. The weight ratio of lignin in polyurethane has a direct impact on the material properties—a higher lignin content translated to better stress–strain behavior (see Figure 3) [40]. The higher stress and strain tolerance and other desirable mechanical properties make lignin-based polyurethane materials appropriate for high-strength applications.

Saito et al. [40] reported the successful synthesis of lignin-based polyurethanes made of telechelic polybutadiene soft segments. Additionally, Da Silva et al. [51] used a kraft lignin precursor to synthesize vanillin and lignin-based polyurethanes, while Cateto et al. [7] synthesized lignin-based polyurethanes using 4,40-methylene-diphenylene diisocyanate (MDI), polycaprolactone (PCL). The successful synthesis of lignin-based polyurethanes from a wide variety of precursors demonstrates that existing synthetic routes can be modified to suit different commercial applications, with or without a catalyst. However, specific adjustments must be made in the synthesis process, including the chemical modification via etherification and esterification reactions [7]. Additionally, oxypropylated lignin materials have been proven useful relative to non-oxypropylated materials in the formation of rigid polyurethane foams. However, there are critical constraints associated with chemical modification. For example, the costs are higher after esterification and etherification; this means that the industrial production of high-value products from lignin precursors is often offset by cost constraints [7], which can be partly offset through the development of new synthetic routes [7,41,51]. The cost constraints reported by Cateto et al. [7] were also acknowledged by Da Silva et al. [51], who argued that cost was not the only constraint.

Other issues relating to the food-versus-fuel dilemma and technical know-how must be resolved before the “novel systems become more competitive against the current industrial alternatives based on petrochemical resources” (p. 1273). The observations made by Cateto et al. [7], Da Silva et al. [51], and Saito et al. [40] illustrate that for lignocellulose feedstock (LCF), bio-refineries are preferred from a sustainability perspective. However, the future development of bio-based products would be catalyzed by conversion products, which offer competitive benefits relative to petrochemical products. This observation is supported by the chemical properties of lignin materials depicted in Table 5.

The physicochemical properties and kinetic properties affirm the suitability of lignin antioxidants in a broad range of engineering and non-engineering applications. However, the formation of the products is dependent on a broad range of factors, including the yield from dopamine polymerization and the polydopamine and copolymers. The antioxidant properties of lignin-based polyurethanes are linked to the presence of an aromatic ring with hydroxyl and methoxy functional groups [50,51]. The functional groups are integral to the oxidation propagation reaction, which is inhibited by hydrogen donation [51]. According to the literature, the performance of lignin polymers was comparable to 2,2-diphenyl-1-picrylhydrazyl (DPPH), and butylated hydroxyl-toluene, among other commercial antioxidant polymers [50].

## 3. Dopamine Polymerization

The self-assembly of polymer molecules has gained significant research attention in research and development because of the versatility of the process, its practicability, and its ability to form products with a broad range of morphologies, including hierarchical assemblies, cylinders, vesicles, and micelles [52]. Polydopamine is a versatile organic molecule that can be deposited onto any substrate at the nanoscale; this means that the material might act as a suitable coat or primer [52,53,54]. The versatile chemistry of polydopamine is linked to the molecule’s ability to form strong covalent bonds [54], physical interactions associated with π-interactions, hydrogen bonding, presence of different surface functional groups including amines, and thiols [54]. However, surface functionalization in isolation does not explain the broad commercial application of dopamine and polydopamine; the material exhibits appropriate antibacterial activities through gelation [54].

The extensive conjugation influences the application of polydopamine coatings in polymers, ceramics, and metals [53]. The extended application of polydopamine in the coating is augmented by extended bio-conjugation, which is integral in the bio-adhesion of protein molecules, adsorption-resistant surfaces, drug delivery systems, contrast agents, and bioadhesives. The adhesion properties of polydopamine are also vital in cardiovascular, diagnostic, and neurotechnology applications [53]. In other cases, the polydopamine coatings have been proven useful in the surface functionalization at the nanoscale to enhance the chemical and electronic properties [55].

The oxidative polymerization of dopamine primarily occurs in the presence of amino-ethyl and catechol groups, which are catalysts for oxidative polymerization [56]—a process that is integral to the formation of PDA nano-coatings and polydopamine coatings [57] through the formation of the aromatic rings of dopamine. Recently, there has been a growing demand for nano-coatings developed using dopamine polymerization techniques; this could be attributed to the capability for secondary modification, generalizability, and simplicity of the synthetic process relative to other techniques [57].

Other unique applications of dopamine polymerization include the electrochemical analysis of dopamine, uric acid, and ascorbic acid using hollow nitrogen-doped carbon microspheres (HNCMS)-based glassy electrodes [58]. The application of dopamine polymerization in biosensors and bioelectronics documented by Xiao et al. [58] was corroborated by Kalimuthu and John [59], who reported successful electrochemical determination of xanthine, ascorbic acid, dopamine, and uric acid using 2-amino-1, 3, 4-thiadiazole (p-ATD)-modified glassy carbon electrodes.

The application of dopamine polymerization in biosensors and bioelectronics and the development of the desired products in manufacturing applications depends on a broad array of factors such as size control of polydopamine nodules and chemical composition of the precursors (carboxylic acid-containing compounds) [60]. The carboxylic-acid-containing compounds introduce an acidic environment, which yields products with unique chemical characteristics compared to dopamine polymerization under basic conditions.

Even though the findings reported by Chen et al. [61] seem to favor acidic dopamine polymerization, basic polymerization techniques have yielded stable products through the customization of the synthesis process. Du et al. [57] observed that the challenges associated with dopamine polymerization under basic environments could be offset by light-triggered regulation of light initiation and termination of dopamine polymerization; this was achieved through the incorporation of small quantities of antioxidant Vitamin C (sodium ascorbate). Vitamin C contributed to the inhibition of the polymerization process under basic conditions—a process that has remained a challenge in traditional synthesis. Du et al. [57] attributed the superior performance to Vitamin C’s ability to delay/inhibit dopamine polymerization and reduce the reactive dopamine quinone. The inhibition process can be halted through UV irradiation. Once vitamin C is exposed to UV radiation, instantaneous dopamine polymerization is achieved. The customization of the process using a natural antioxidant and UV radiation helps to explain why it was feasible to attain optimal dopamine polymerization under basic conditions through a facile, scalable, and environmentally benign process.

Fichman and Schneider [54] reported a facile synthetic route for dopamine polymerization under basic conditions in the absence of Vitamin C. Optimal polymerization was achieved in this case through gelation of 1 wt% MAX1 peptide; this experiment was conducted in the presence of 10 mM dopamine at neutral pH [54], and molecular oxygen. The process resulted in the spontaneous polymerization of dopamine. The temperature was adjusted to room temperature to trigger a hydrophobic effect, which was instrumental in promoting MAX1 assembly. Considering that the final product exhibited a 66-fold improvement in mechanical rigidity relative to other materials synthesized using alternative synthetic routes [54], sodium ascorbate is not a prerequisite.

The reports of the positive synthesis of polydopamine and dopamine under basic and acidic conditions by Chen et al. [61], Du et al. [57], Fichman and Schneider [54], Kwon and Bettinger [53], and Qui et al. [52] do not address other emergent challenges such as the relationship between film thickness and solution pH, dopamine concentration and self-polymerization time optimization. The critical requirements for the process underscore the need to select a suitable pH range for the controlled synthesis of materials with the desired film thickness, mechanical rigidity, and industrial/biological performance.

### 3.1. Polydopamine and Copolymers

**Figure 4:**
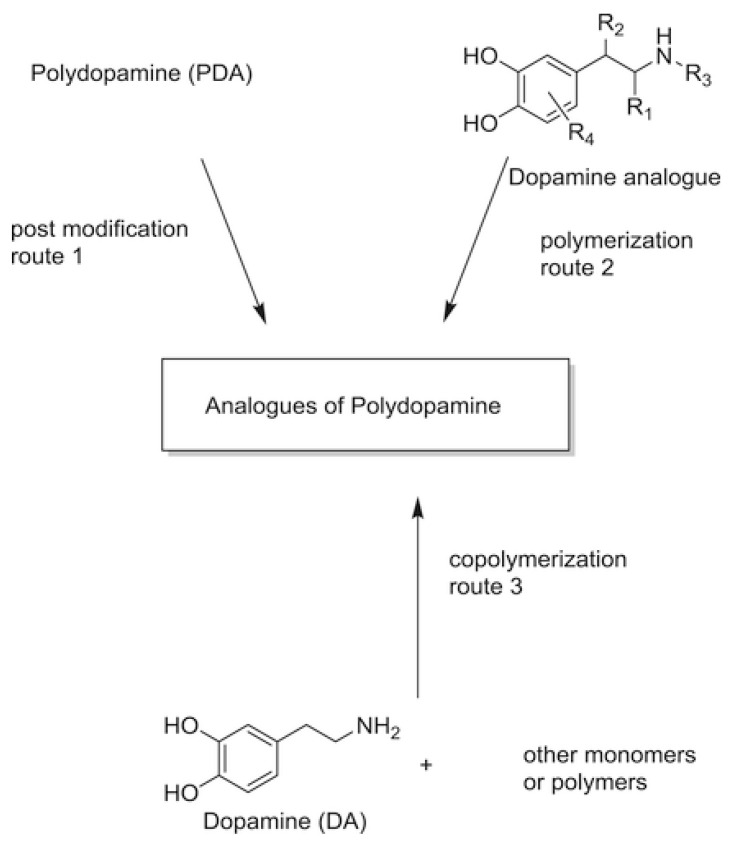
Polydopamine polymerization from different analogs [62].

The chemistry of polydopamine (PDA) copolymers predicts the synthesis of derivative materials and promising applications in biomedicine, environmental, and energy applications. The application of PDA is predicted by the presence of the different functional groups (azido, alkyl-thiol, amino, carboxyl derivative groups, and carboxyl and alkyl groups) and the synthesis process [62]. There are three feasible mechanisms for polydopamine synthesis from derivatives, namely oxidative copolymerization of the mixtures (comprising of copolymers and monomers), and oxidative polymerization of DA analogs with additional functional groups (see Figure 4).

#### Melanoidins

Melanoidin compounds are byproducts of the Maillard reaction and the reduction of amino acids, sugars and proteins during food preservation and processing [61]. In most cases, the compounds are drawn from coffee processing—the roasting of coffee bean components results in the generation of brown-colored nitrogenous compounds with high molecular weight [62], referred as melanoidins.

## 4. Polymerization of Inulin

Inulin is categorized as a fructan carbohydrate, which functions as a primary ingredient in industrial food processing. Inulin hydrogels have been employed as carriers for colonic drug targeting; such processes are fundamental to prebiotic fermentation, chocolate, and cheese production [63,64,65], as noted in the preceding sections. From a nutritional perspective, the functional properties of inulin and health beneficial effects include prebiotic effects, high dietary fiber content, and lower calorie value [65]. The physiological benefits linked to the consumption of inulin-rich foods are presented in Table 2. In industrial food production, inulin has been employed in fat replacement as a bulking agent [65], stabilizer, and fat replacer in cheese production. The observations made by Karimi et al. [65] are in agreement with Aidoo et al. [63], who reported the incorporation of inulin in sugar-free chocolate production (inulin functions as an alternative sweetener), and Guimarães et al. [64] research on inulin’s role in prebiotic fermentation (especially in beverage stabilization). In other cases, inulin has been proven useful in the retro-gradation and gelatinization of wheat starch [66].

The potential applications of inulin are not confined to food production, given that inulin has been proven to be an ideal in vitro drug carrier of lignin derivatives. Vervoot et al. [67] noted that inulin was an ideal carrier for colonic drug targeting [67]; this is achieved through the surface functionalization of inulin with N, N, N′, N′-tetramethyl-ethylenediamine, and ammonium persulphate [67]. Colonic drug delivery is critical to the treatment of colon cancer and metabolic/bowel complications. Similar to Vervoot et al. [67], Kumar et al. [68] noted that inulin was a suitable bioactive polymer for pathogen-mimicking vaccine delivery systems. The biomedical applications of inulin in targeted drug delivery offer promising prospects in the treatment and management of life-threatening conditions, including cancer, Ebola, HIV/AIDS, malaria, and tuberculosis. The biomedical application of inulin is mediated by free-radical polymerization—a process that contributes to the incorporation of vinyl functional groups on the surface of inulin [67].

Beyond industrial applications, the polymerization of inulin has broader implications on the health of mammals. Wada et al. observed that the fermentation of indigestible carbohydrates in the diet by bowel microflora predicts the formation of short-chain fatty acids [69]. Acetate, propionate, and butyrate are the primary short-chain fatty acids (SCFA) derived from the digestion and fermentation of undigested carbohydrates and inulin in the human colon [70].

The formation of both short-chain and long-chain fatty acids has long-term effects on bowel physiology. The observations reflect long-standing beliefs and evidence concerning the relationship between improved health outcomes and high-fiber diets, causal epidemiological evidence, and metagenomics studies linking metabolic diseases to variations in the gut microbe and molecular signaling [71].

Even though there is growing use of inulin in biomedical and food-related industries, the extent of application depends on a broad range of factors, including the polymerization of inulin; this underscores the need for customized synthetic processes, including the reaction between N,N-dimethylformamide with glycidyl methacrylate in the presence of a catalyst (4-dimethylaminopyridine) [67]. The polymerization of inulin is integral to industrial application, primarily in the food and biomedical sectors, but there are critical technical constraints that impede successful polymerization.

## 5. Enzyme-Catalyzed Polyphenol Polymerization

Similar to the antioxidants for food production and biopharmaceutical applications, enzyme-catalyzed polyphenol polymerization reactions have a broad range of practical applications, including antioxidant, antibacterial, anticancer, cardioprotective, and neuroprotective properties [72]. However, the progression of most reactions is impeded under natural conditions; this justifies the need for enzyme-mediated reactions to attain the desired effects. In a recent study, Oliver et al. [72] noted that the impressive therapeutic activity of polyphenols in therapeutic agents and other clinical applications could not be realized without proper adjustment of the production processes and chemical properties. Under natural conditions, polyphenols exhibit poor membrane permeability, rapid metabolism, and poor bioavailability and UV degradation. The challenges have been partly resolved through amidification, esterification, free-radical grafting [73], step-growth, free radical, and enzyme-catalyzed reactions (direct polymerization of polyphenol monomers), and enzyme grafting-mediated conjugation with macromolecules.

**Figure 5:**
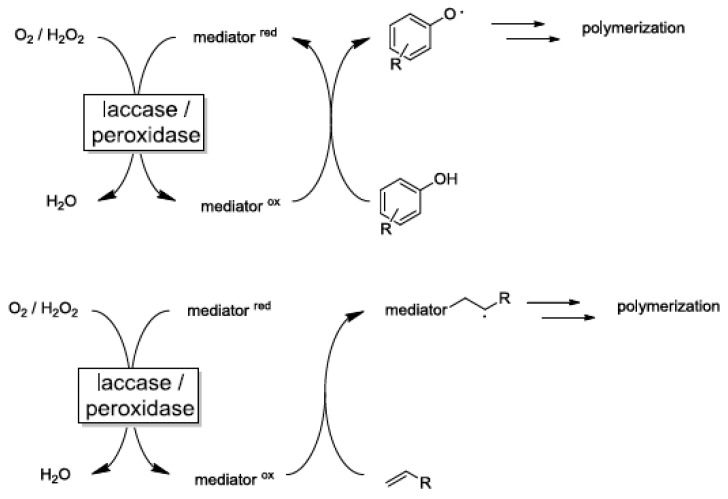
Illustration of laccase and peroxidase mediator systems (PMSs) in polymerization reactions [76].

Even though multiple techniques have been proposed to improve the industrial application of polyphenols, enzyme grafting and catalyzation of the reaction were given preference given polyphenol polymerization under natural conditions is constrained by the reaction mechanisms and chemical composition of the reactants, which impede synthesis using conventional methods [74,75]. The forward polymerization process is augmented by the inclusion of enzymes. The utility of laccase (an enzyme) in catechol, resorcinol, and hydroquinone selective polymerization was confirmed by Sun et al. [75] (see Figure 5). The process was phased; the initial phases entailed the formation of quinone intermediates through laccase catalysis, followed by oxidation and formation of covalent bonds. In the subsequent steps, carbon-carbon (C-C) and ether bonds were formed, linking catechol units, resorcinol, and hydroquinone units.

A key benefit associated with the reaction process is the formation of reaction products under mild conditions and the elimination of toxic byproducts such as formaldehyde [74]. The merits of the laccase-catalyzed polymerization process are offset by the complexity of the reaction mechanism [76]. Hollman and Arends [76] argue that even though catalysts are critical to the progression of reactions, which are unfeasible using conventional methods, there are practical constraints such as unfavorable steric interactions between the polyphenols and the active sites within the enzyme. Alternatively, the suitability of the enzymes in reaction processes could be impaired by unfavorable redox reactions. Since enzymes in isolation do not address the challenges and limitations associated with the synthesis of new products, alternative processes must be developed to improve the yields in enzyme-catalyzed polyphenol polymerization reactions.

Other experiments have reported the practical benefits of using alternative enzymes, primarily hydrolases, to catalyze bond cleavage reactions and the reverse action of hydrolysis [74]. Similar to laccase hydrolyses, they catalyze polyphenol polymerization via bond-forming reaction. However, hydrolyses are suitable in oxidative polymerization reactions that are specific to peroxidase and laccase catalysts in the presence of phenol derivatives. Other factors that inform the choice of enzymes for polyphenol polymerization include inhibition of laccase-initiated polymerization and peroxidase-initiated polymerization and the use of molecular oxygen in place of hydrogen peroxide [76]. The focus on molecular oxygen was validated by the following considerations.

Even though hydrolyses, laccase, and peroxidase polymers have proven to be useful in polyphenol polymerization, the concerns associated with polyphenol polymerization should be addressed. Various mechanisms were proposed by Hollman and Arends [76] to address the catalyst-related problems; these include the ingenious use of a mediator as a radical transfer catalyst. However, the radical is not incorporated into the end product. The feasibility of this reaction has been demonstrated in the use of phenothiazines to address constraints associated with the sterically hindered/congested phenols and consequently catalyze the polymerization process. In other cases, the production constraints can be offset by the incorporation of ABTS or transition metal ions, particularly Mn^2+/3+^, to facilitate the pyrrole polymerization process. If transition metal ion species and ABTS prove less useful, polyoxometallates can be employed. The complexities of polyphenol catalysis underscore the challenges that impact the synthesis and application of sustainable and natural monomers and antioxidants in food packaging, biopharmaceuticals, and agricultural applications.

The synthesis-related constraints raise fundamental questions about the commercial exploitation of naturally occurring antioxidants such as tocotrienols and tocopherols [1,2,3], phenolic compounds, carotenoids [4]. Even though polymerization of natural polyphenols is impacted by the generation of formaldehyde byproducts, unfavorable steric interactions between the polyphenols and the active sites within the enzyme, inhibition of laccase-initiated polymerization, the process offers practical benefits relative to the use of artificial antioxidant molecules such as α-lipoic acid, N-acetyl cysteine, melatonin, gallic acid, captopril, taurine, catechin, and quercetin [5], or the replacement of catalysts with alternative processes such as amidification, esterification, free-radical grafting [73], step-growth, and free radical-catalyzed reactions to improve the percentage yield (see Table 6).

The outcomes documented by Hollman and Arends [76] validate the polymerization of polyphenols, quercetin, and other organic molecules with suitable antioxidant properties. A critical challenge concerns the shortcomings and tradeoffs of existing polymerization processes.

## 6. Antioxidant Quercetin Polymers

The polymerization of quercetin is part of a broader effort in recent years to enhance the performance of natural and synthetic antioxidant polymers via covalent insertion of antioxidant species (such as vitamins, curcumin, and quercetin) into polymeric chains [77]. The polymerization products have demonstrated appropriate properties in food packaging (as preserving agents) [78], biomedical and pharmaceutical applications, especially in the production of antioxidant-laden nanoparticles for cancer treatment [77,79,80], or contact lenses and hemodialysis membranes. In both applications, the potency of the antioxidants is contingent on successful polymerization. A distinct advantage of bioconjugates is the exploitation of unique benefits of different bio-conjugates, the slow rates of macromolecular system degradation, and better chemical and cellular stability.

Quercetin is a plant-derived aglycone flavonoid with antioxidant properties that are suitable in the food packaging industry and the production of targeted therapies for cancer, heart, liver, and lung complications [78,80,81]. The primary sources include tea, red wine, and common fruits and vegetables such as onions, berries, apples, red grapes, broccoli, and cherries [80]. The antioxidant properties influence medical and nutritional applications as nutraceutical/nutritional supplement to boost the immune system and elevate protection against cardiovascular and lung conditions, osteoporosis, and tumor growth [80,81]; this is achieved through the neutralization of reactive oxygen species and the mediation of the transduction pathways, enzymatic activity, and function of glutathione. The specific mechanisms for the prevention of liver damage include the inhibition of the cellular activation of various signaling pathways, including NF-κB and MAPK [80]. Moreover, the antioxidant inhibits the release and expression of apoptosis-related proteins triggered by LPS/d-GalN, suppresses oxidation marker-mediated production of LPS/d-GalN. Following the inhibition of the mechanisms listed above, quercetin catalyzes the antioxidant signal transduction pathways (particularly Nrf2/GCL/GSH) and other processes that increase the concentration of glutathione in the cells [79].

The chemical structure and properties of quercetin contribute to its bioavailability and solubility in cellular fluids and moderation of biochemical pathways. However, the commercial exploitation of the antioxidant properties of quercetin depends on the success of the polymerization process, which can be tailored to generate bioactive compounds such as template quercetin (QCT) nanoparticles for free-radical scavenging. The cellular function of the QCT nanoparticles was demonstrated through material characterization using transmission electron microscopy (TEM), dynamic light scattering, H-NMR spectroscopy, Fourier-transform infrared spectroscopy, and UV-Visible spectroscopy [80]. The demonstrated biopharmaceutical application of oxidation-triggered self-polymerization quercetin in the production of targeted therapies by Xu et al. [80] is in line with the findings of Sunoqrot et al. [79]. The latter study documented the successful bio-inspired polymerization of quercetin—a process that resulted in the synthesis of cancer therapies. The nanotechnology-based quercetin–curcumin therapeutics exhibited superior cytotoxic behavior against cancer tumors. However, the superior biological function was dependent on precision in the synthetic process.

In the current case, the quercetin–curcumin-loaded nano-medicine was synthesized in the presence of curcumin and thiol-terminated poly (ethylene glycol) (PEG)-mediated surface functionalization; this was integral in facilitating steric stabilization in a single reaction step. The reactants were exposed to dimethyl sulfoxide [79]—a universal solvent to obtain a homogenous solution. The next polymerization steps entailed the gradual addition of water. The single-step synthesis process preferred by Sunoqrot et al. [79] was confirmed to be efficient by Pouci et al. [77], who noted that the one-step synthesis route was integral to the synthesis of polymer antioxidant conjugates (that are less susceptible to degradation) without emitting toxic byproducts.

Even though Sunoqrot et al. [79] documented successful synthesis and clinical application of quercetin–curcumin-loaded nanoparticles for targeted cancer drug synthesis, the approach does not align with Zhang et al.’s [81] research on the antioxidant properties of quercetin. In contrast to Sunoqrot et al. [79], Zhang et al. [81] advocated the use of quercetin based on its greater reduction potential relative to curcumin. The reduction potential helps to predict the total antioxidant capacity (TAC), which is fourfold higher for quercetin compared to curcumin. The unique chemical and biological properties help explain the cellular action against LPS-induced reactive oxygen species. Considering that experimental data predict clinical applications, further research is necessary to assess the effectiveness of quercetin only and quercetin–curcumin-loaded nanoparticles for targeted cancer tumor treatment. The need to compare antioxidants’ potential benefits in combined or individual treatments is consistent with the research of Zhang et al. [81].

**Figure 6:**
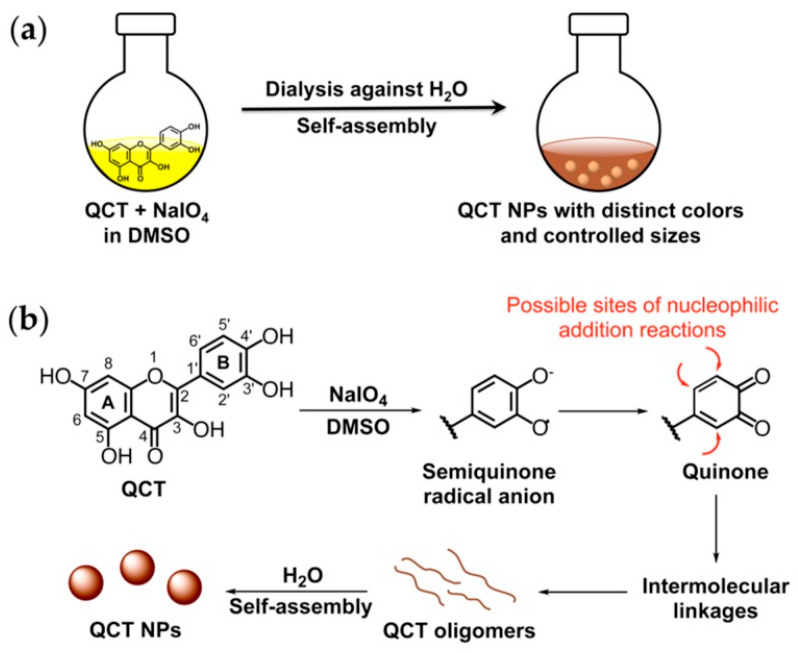
Synthesis process for template quercetin (QCT) nanoparticles [80] (**a**) Schematic of synthesis and (**b**) Potential chemical pathways of synthesis.

The biomedical applications of quercetin documented by Xu et al. [80] are but a microcosm of the potential industrial applications considering that quercetin is indispensable in the food production industry, where it is incorporated as an additive in bio-polyether (PEO), bio-polyester (PLA), and commercial starch-based polymer (Mater-Bio) [78]; this depends on the extent of photo-stabilization and artificial photo-stabilization. The oxidation-triggered self-polymerization is depicted in Figure 6. The illustration demonstrates the role of incubation, oxidizing agent, and universal organic solvents in the synthesis of nanoparticles.

The impact of quercetin additives on the tensile strength, Young’s Modulus, and elongation at break of bio-polyether, bio-polyester, and commercial starch-based polymer (Mater-Bio) is depicted in Table 7. The data demonstrate the influence of quercetin on stabilized and un-stabilized systems. The mechanical testing outcomes showed a significant improvement in the elastic modulus, tensile strength, and elongation at break after stabilization with quercetin additives (Q): Cyasorb^®^ and synthetic Light Stabilizers (LS). In particular, a 5 wt% increase in elongation at break and the tensile strength resulted in a 10–20% improvement in the elastic modulus, tensile strength, and elongation at break. However, the microscale changes in the mechanical properties were attributed to different mechanisms.

On the one hand, the changes in the elongation at break of the stabilized bio-polyether, bio-polyester, and commercial starch-based polymer have been linked to the plasticizing effects triggered by the low MW stabilizing molecules such as quercetin [78]. On the other hand, the improvements in the tensile strength and elongation at break have been linked to higher rigidity of the stabilized films in relation to the machine direction [78]. The contrasting changes at the micro scale show that quercetin has unique effects on biofilms for food packaging—a phenomenon that introduces new challenges and benefits in the polymerization of quercetin for industrial applications. On a positive note, the higher orientation of the macromolecules in the presence of the plasticizing agent increases the suitability of quercetin in food packaging. It is deduced that other challenges could be addressed through innovations in polymerization.

The improvements in elastic modulus, tensile strength, and elongation at the break following stabilization with Q and LS by Morici et al. [78] are significant considering that the most common forms of quercetin polymerization are inspired by nature. The polymerization of polyquercetin offers further practical opportunities for developing customized therapeutics that influence glutathione (GSH) and enzymatic activity, moderate the signal transduction pathways, and diminish the availability of reactive oxygen species (ROS).

### Polyquercetin and Quercetin Copolymers and Antioxidant Properties

Similar to quercetin, polyquercetin has superior antioxidant properties, depending on the synthetic route and ingredients. A facile and scalable micro-emulsion polymerization/crosslinking technique was developed by Sahiner [82], involved reacting quercetin molecules with glycerol diglycidyl ether (GDE) in the presence of l-α lecithin and cyclohexane, which act as the surfactant and organic phase, respectively. The process was proven to be useful in the synthesis of polyquercetin and quercetin copolymers with customizable antioxidant properties. In contrast to the methods proposed by Sahiner [82], Pivec et al. [83] explored an alternative technique comprising 0.5 g of flavonoid rutin (RU) hydrate in ultra-pure water. The mixture was subjected to enzymatic polymerization through the incorporation of Trametes Versicolor-based laccase (500 mg). The mixing was performed at room temperature, away from UV light for 24 h, after which ammonium sulfate was incorporated to enhance the precipitation process; this was followed by centrifugation, removal of the supernatant solution, and dialysis. A comparison of the two techniques shows that the microemulsion polymerization/crosslinking technique developed by Sahiner [82] was more facile and scalable compared to the lengthy procedures undertaken by Pivec et al. [83]. The focus on the synthetic route is grounded on the chemical structure–antioxidant activity relationship discussed in the preceding sections [77,78,79,82,83,84,85].

In contrast to quercetin molecules, which have found broad applications in targeted drug delivery and food packaging, polyquercetin has mainly been used as an electrode component for glass carbon electrodes modified by multi-walled carbon nanotubes; this was the case in studies conducted by Ziyatdinova et al. [84,85]. In both studies, the oxidation potentials of polyquercetin influenced the chronoamperometric determination of the antioxidant capacity and the quantification of gallic acid, catechin, and epigallocatechin gallate (EGCG) [84,85]. The variable industrial applications of quercetin and polyquercetin demonstrate the influence of polymerization and the choice of the crosslinking method.

## 7. Antioxidant Terpene Polymers

Similar to polyphenols, tocotrienols, tocopherols, quercetin, α-lipoic acid, N-acetyl cysteine, melatonin, polyquercetin, gallic acid, captopril, taurine, vitamins, and catechin terpenes are antioxidant polymers synthesized via natural and artificial polymerization processes [86,87,88,89,90]. However, the synthesis and commercial application of terpenes as antioxidants in food packaging and biomedical industries has attracted less research interest compared to polyphenols, vitamin C and D, and quercetin polymers—a phenomenon that is linked to the unique function and features of different terpenes compounds (D-limonene, β-ionone, geraniol, eugenol, myrcene) [91]. For example, each of the listed terpenes functions as an ideal insecticide (in citral mixtures) due to the antibacterial and antifungal properties (see Table 8); there are no notable side effects on human health given the diminished neurotoxicity risks.

Despite the potential for application in various manufacturing applications, there has been minimal industry interest in terpene and terpene derivatives. Winnacker et al. [88] attributed this phenomenon to inadequate understanding of how terpenes could be combined with other natural and synthetic polymers to augment material characteristics (such as tensile strength, photo-degradation, elongation at break, antimicrobial properties, and biocompatibility) [88]. Moreover, even though the feedstock is cheap and widely available, there are gaps in knowledge concerning effective chemical processes to reduce material costs, and determining potential new applications for terpene derivatives and monomers remain underexplored [88].

The most widely applied procedures include radical initiator-free addition and solvation of thiols to terpenes such as R +/− and S−/− limonene and -β-pinene; chain and step-growth polymerization techniques involving acrylic monoterpenes myrcene (7-methyl-3-methylene-1,6-octadiene) and ocimenes and Bayer–Villiger oxidation of menthone into methide [89]. The application potential of terpenes includes the synthesis of terpene/fatty acid-based polyesters for industrial applications [89]. The reaction parameters, including conversion efficiency for polyester synthesis via copolymerization of propylene oxide (PO), are depicted in Table 9 [88].

In other cases, terpenoid-based polymeric resins have been employed in stereolithographic 3D printing [87] and as starting materials in the production of sustainable polymers and advanced materials [88]. The renewed industry attention on terpenes and their derivatives is validated by the progress made in controlled polymerization, interesting structures, and the broad scope of industrial application, renewability, and abundant feedstock.

### Polylimonine Synthesis and Properties

The synthesis of polylimonine has practical implications on the production of terpenes, given that the former classes of chemical compounds are optically active terpenes, which exhibit antioxidant behavior [92]. Commercial applications of limonene and polylimonene include food preservation, enhancing the flavor and aroma of foods [93]. A fundamental constraint in the development of polymeric products globally is the recyclability of the precursor and the ecological effects; this remains a priority concern in polylimonene synthesis, considering that most precursors are sourced from fossil fuels [94]. In light of this challenge, the synthesis of non-toxic and renewable plastics is a priority for manufacturers. Such plastics would be ideal substitutes for existing non-renewable polymers. Traditionally, ideal precursors for renewable polymers are starch, essential oils, and wood. However, there have been growing research interests in the production of renewable and non-toxic plastics from terpenes.

Terpenes are naturally found in citrus plants and essential oils. Other sources of terpenes include tea, thyme, cannabis, Spanish sage, and citrus fruits (lemon, orange, mandarin) [91]. Beyond the ubiquity of the precursors, terpenes (D-limonene in particular) are ideal based on their chemical structure and the presence of isoprene units; these units are key enablers for addition-based polymerization processes. The choice of D-limonene as a precursor in the current case is supported by the worldwide production capacity [94] and compatibility with microbial mediated synthesis processes, including PMD, Codon optimized MVK, and PMK (see Table 10).

The biotechnology-based production of limonene from microorganisms does not address production-related constraints, including the molar mass loss/lack of sufficient molar mass, selection of an acceptable monomer for conversion. Most of the precursors for terpenes have practical drawbacks and limitations—a factor influencing the choice of orange peel-based precursors—a precursor proven useful in the synthesis of polylimonene through recycling polymerization [94]. Other sources of terpenes include tea, thyme, cannabis, Spanish sage, and citrus fruits (lemon, orange, mandarin) [91]. Beyond the ubiquity of the precursors, terpenes (D-limonene in particular) are ideal.

Under standard conditions, Coelho and Vieira synthesized polylimonene by placing D-limonene in a solution containing Ziegler-Natta catalysts and Lewis acids in the first stage. The molar mass of the mixed species was below 1000 g/mol [94]. The second stage entailed the initiation of free-radical polymerization using benzoyl peroxide. The concentration of the free radical initiator is adjusted accordingly to attain the desired conversion efficiency. In the current case, the desired conversion efficiency was 12% [94]. The reduced copolymerization rates demonstrate there is an inverse link between the D-limonene ratio, molar masses, and concentration of styrene, methyl methacrylate, and n-butyl acrylate).

## 8. Antioxidant Random Copolymers

The synthesis of antioxidant copolymers such as dual-function amphiphilic random copolymers is critical to the suppression of medical-related infections through Fe ^2+^ chelating ability and supra-macromolecular re-arrangement and morphological changes in hydrous environments and biocompatibility [95]. The polymerization of the tertiary amine-containing cationic monomer with hydroxyl-tyrosol yielded copolymers with ideal amphiphilic balances, molar ratios, and functional groups (especially the aromatic rings) for biomedical device coatings. In other cases, polypropylene (PP) antioxidant random copolymers have been proven useful in hot water applications in the presence of nucleating agents. Even though the utility of nucleating agents was not demonstrated by Taresco et al. [95], Grabmann et al. [96] argued that the compounds contributed to the modification of the crystalline structure—a factor that translated to higher thermal stability, mechanical strength (toughness and creep); this was observed in β-nucleated PP-R grades. In contrast, non-nucleated grades with α-crystal structure did not exhibit improvements in thermal and mechanical properties.

The utility of antioxidant random copolymers transcends biomedical devices to encompass food packaging applications, but the suitability of the materials for such applications is offset by the rapid depletion of antioxidants and chemical changes induced by microwave heating. Shahidi [97] observed that the viability of polymers for food preservation depends on the ability of natural antioxidants, additives, and preservatives to prevent rancidity—a process that impacts the odor and sensory appeal of foods.

Alin and Hakkarainen [98] explored the suitability of propylene-ethylene copolymer (PP-C), propylene-ethylene random copolymer (PP-R), and polypropylene homopolymer (PP) in plastic packaging containers labeled as microwave safe. The microwave testing of the polymers established the following. First, excessive heating (and subsequent analysis using microwave-assisted extraction (MAE) and HPLC) triggered the migration of the antioxidant molecules, particularly Irgafos 168 and 100 from the propylene-ethylene copolymer (PP-C), propylene-ethylene random copolymer (PP-R), and polypropylene homopolymer (PP) [98]. The migration resulted in unequal distribution of the antioxidants diminishing the utility of the polymer packaging materials in food packaging. A notable observation was the influence of polypropylene materials on the migration rate of the antioxidants and the inverse relationship with crystallinity. Better migration resistance was noted in the polypropylene homo-polymer (PP) potentially due to higher crystallinity [98]; this pattern remained unchanged despite contact with fatty acid stimulants. The influence of acetic acid and ethanol/iso-octane concentration on the rate of migration is depicted in Table 11.

In light of the preliminary observations made by Alin and Hakkarainen [98], polypropylene homo-polymer (PP) is an ideal alternative compared to propylene-ethylene copolymer (PP-C), propylene-ethylene random copolymer (PP-R), considering that the migration of antioxidants promotes toxicity and emergence of undesirable flavors [99]. A fundamental constraint moving forward was the development of packaging materials that are suitable and resistant to antioxidant migration, regardless of the fatty acid concentration in food. The experimental data shows that the composition of the food items, exposure to heat, and microwave energy predicts the chemical composition of antioxidant polymers for food packaging.

### 8.1. Gallic Acid Grafted Polymers

Similar to lignin, polyphenol, and quercetin polymers, gallic acid grafted polymers are ideal for targeted drug delivery, packaging, and commercial, industrial applications. Cho et al. [100] documented successful synthesis and characterization of gallic acid-grafted-chitosans, which demonstrated superior antioxidant scavenging ability against various free radicals such as hydrogen peroxide (93% effectiveness) and 2,2-diphenyl-1-picrylhydrazyl (92% effectiveness) at low concentrations (50 μg/mL) [101]. The outcomes affirm the superiority of gallic acid grafted copolymers in free radical scavenging. In other studies, gallic acid grafted chitosan-casein phosphopeptide nanoparticles have demonstrated superior anticancer and antioxidant properties under simulated gastrointestinal conditions [102]. The observations made by alternatively, the incorporation of chitosan on resveratrol via free radical-induced grafting proven effective in the synthesis of resveratrol modified species [73]. Cho et al. [100] contrast with Curcio et al. [79], who documented successful free radical-induced grafting of gallic acid-chitosan and catechin-chitosan conjugates for antioxidant applications.

Considering that the in-vitro models were confined to the two free radicals (2,2-diphenyl-1-picrylhydrazyl, and H_2_O_2_, it remains unclear whether gallic acid-grafted-chitosans would retain the high antioxidant scavenging ability at higher concentrations. The outcomes documented by Cho et al. are in agreement with Spizzirri et al., who documented successful synthesis of antioxidant−protein conjugates (gallic acid (GA) and catechin (CT) attached on gelatin) via grafting reaction [103]. Similar to gallic acid grafted polymers, the Gallic Acid-Catechin-Gelatin copolymers exhibited superior scavenging ability against linoleic acid peroxide hydroxyl radicals and 2,2′-diphenyl-1-picrylhydrazyl.

### 8.2. Resveratrol Conjugated and Grafted Polymers

Resveratrol exhibits comparable antioxidant activity as quercetin, polyphenols, Vitamin C, and E, phenolic compounds, carotenoids, α-lipoic acid, melatonin, gallic acid, and N-acetyl cysteine, captopril. However, the material is superior to other antioxidants based on its broad biological activity, including anticancer, anti-inflammatory and chemoprevention properties, and rapid metabolism within the body due to its shortened half-life [104]. The mechanical, biological, and chemical properties can be enhanced through modifications in the synthetic routes. For example, Siddalingappa et al. [104] reported the successful synthesis of Resveratrol-PEG ethers via alkali-mediated etherification. The plasma stability of the moieties was augmented by aggregation behavior and polymer hydrophobicity, and the antioxidant demonstrated ideal antioxidant behavior in mice models. Further customized changes were viable through carbodiimide coupling reaction-mediated surface and chemical modifications with α-methoxy poly (ethylene glycol)-co-polylactide succinyl ester resveratrol, MeO-PEG succinyl ester resveratrol, and α-methoxy-ω-carboxylic acid poly(ethylene glycol) succinylamide resveratrol [104].

## 9. Antioxidant-Polysaccharides Graft Polymers

Polysaccharide graft polymers play an integral role in biomedical (targeted drug delivery systems) and commercial engineering applications given their unique properties and wide range of sources (microbial and natural). In contrast to other precursors, polysaccharides are cheap and widely available [105]. Moreover, the materials have comparable properties as antioxidant terpene polymers in terms of biodegradation ability, water solubility, and non-toxicity. Grafted polysaccharides have attracted significant research attention based on a broad scope of application, chemical stability, efficiency, biocompatibility, tailored properties, and biodegradation ability. Recent advances in drug delivery research have demonstrated that the rate of in vivo drug delivery could be predicted by the extent of crosslinking and grafting [105]. The enhanced functionality is linked to chemical bonding between the grafted polymer chains and polymeric substrate. The free radicals are introduced onto the backbone structure through irradiation and chemical initiation.

Despite the promising material prospects, the polysaccharide graft polymers have not conclusively addressed challenges associated with traditional drug delivery systems, such as sustaining the desired drug dosage in tissues for an extended period (length of treatment). However, there are promising prospects that the uncontrolled drug release kinetics would be resolved through better absorption rates [105]. The progress made in targeted drug delivery by Pal and Das [105] is in line with Lemarchand et al. [106] research on polyester-polysaccharide nanoparticles for drug delivery systems. In particular, the nanoparticles graft polymers from poly (D, L-lactide) or poly(ε–caprolactone) (PCL) demonstrated ideal pharmacokinetic behavior linked to the synthesis route—emulsion solvent evaporation. The material exhibited superior stability with or without emulsions, and strong amphiphilic properties, which eliminated the need for surfactants.

The emulsion solvent evaporation technique’s ability to influence the targeted drug delivery is comparable to RAFT (reversible addition-fragmentation chain transfer polymerization), reversible deactivation radical polymerization, and atom transfer radical polymerization; these two procedures were employed by Garcia-Valdez in the modification of polysaccharides to attain ideal control over the molecular weight distribution, and carefully control the macromolecular shapes, and sizes [107]. From an engineering perspective, the experimental outcomes reported following the adoption of different synthetic routes introduce unique challenges because the outcomes were based on laboratory-scale models; there are concerns that the procedures could lead to different outcomes in large experiments. The highlighted concerns could be resolved through advances in synthesis technologies, including lab-on-a-chip.

## 10. Poly (Trolox Ester) Polymers

Poly Trolox ester polymers are useful antioxidants in human cells based on their ability to relieve oxidative stress in injured cells and resolve iron particle toxicity at the nanoscale [107,108]. Even though there is a broad class of compounds that exhibits antioxidant behavior (including α-lipoic acid, N-acetyl cysteine, melatonin, gallic acid, captopril, taurine, catechin, and quercetin), the biological function of most is impaired by oxidative stress-linked biological incompatibility at the nanoscale [108]. Wattamwar et al. claimed that poly Trolox ester polymers were able to overcome these barriers by inhibiting the response of free radicals while retaining higher compatibility with medical devices. In contrast to quercetin, lignin, and polydopamine, poly Trolox ester polymers are a class of synthetic analogs of vitamin E (with superior hydrophilic, tunable particle sizes, antioxidant properties) [108]. The materials can be synthesized via a facile single emulsion technique; the nanoparticle concentration is varied over time to achieve the desired nanoparticle sizes.

Resolving iron particle toxicity is a priority in biomedical applications, given that the risk is elevated by the frequency of medical diagnosis and treatment (iron supplements for anemia and MRI contrast agents). Iron particle toxicity was mitigated through targeted delivery of functionalized poly Trolox ester polymers to the platelet endothelial cell adhesion molecules (PECAM-1) [101]. In theory, the modification of the pro-oxidant and antioxidant activity of poly Trolox ester polymers could be applied in virtually all high-oxidative-stress environments within the cells. However, in reality, this might not be feasible considering that poly (Trolox ester) nanoparticles’ ability to induce/inhibit cellular oxidative stress depends on advanced programming of the molecules, based on knowledge of the molecular compounds being oxidized and the cumulative oxidative status of the cells [6]. If the listed variables are not known, the cellular function of poly (Trolox ester) nanoparticles might be impaired. Such complexities limit the clinical application of Trolox esters.

## 11. Polymerization of Vitamins

Reversible addition-fragmentation chain transfer (RAFT) is suitable for the Vitamin C and B2 mediated polymerization of synthetic monomers such as methacrylates, acrylates, and acrylamides under LED irradiation. The efficiency of the polymerization process is contingent on the optimization of the irrational wavelength and oxygen. Zhang et al. linked the high throughput in vitamin-mediated polymerization to the low reaction volume platform, molecular weight distribution, low reaction volumes and the concentrations of Vitamin B_2_ and C [109]. The costs of the synthetic process can be offset by the inclusion of off-the-shelf vitamin supplements in place of reagent/analytical grade Vitamin C and B_2_. The free radical-initiated chain polymerization of biomolecules has critical implications on disease prevalence, given that the progression of Alzheimer’s disease and other neurodegenerative diseases can be linked to free radical damage of cells. On a positive note, this process can be offset by anti-oxygenic nutrients, such as vitamin-rich (C, E and co-enzyme Q) fruits and vegetables, which offer protective benefits against free radical damage [110]. The polymerization of compounds with complex architectures has been augmented by advances in technology, particularly photo-induced electron/energy transfer–reversible addition-fragmentation chain-transfer (PET-RAFT) polymerization.

The PET-RAFT process is remarkably superior relative to RAFT in isolation due to higher oxygen tolerance [111]. However, other issues remain unresolved, including the experimental testing of oxygen tolerant species. Most of the documented experiments were conducted in water—a factor that limits the utility of the process in the presence of organic solvents. The initiation of vitamin polymerization in the presence of organic solvents is critical in biological processes, given that vitamins and biological molecules comprise organic functional groups. The constraint was partially resolved by Gormley et al. [109], who documented successful polymerization of oxygen resistant compounds in organic solvents, dimethyl sulfoxide (DMSO). Nonetheless, it is of note that the initiation of the process requires a zinc tetraphenyl porphyrin (ZnTPP) catalyst. From an industrial and commercial perspective, the additional costs linked to catalysis pale in comparison relative to the additional benefits associated with successful PET-RAFT in organic solvents. The findings documented by Zhang et al. [110], Tappel et al. [111], and Gormley et al. [109] demonstrate the excellent potential of oxygen tolerant PET-RAFT and RAFT vitamin-based photo-initiation mechanism in biological applications.

## 12. Conclusions

The published literature has demonstrated the antioxidant properties of various naturally occurring compounds, such as quercetin, polyphenols, vitamins, and synthetic molecules including α-lipoic acid, N-acetyl cysteine, melatonin, gallic acid, lignin, captopril, taurine, and catechin; the free radical scavenging abilities predicted the potency of the antioxidants (hydrogen bonding within the ether O_2_ atoms in peroxide radical) and the location of bulky tert-butyl functional groups. The extraction of antioxidants from agro-wastes generates high-quality phenolic antioxidants. On the downside, the benefits are offset by low yields and negative ecological effects, as well as synthesis-related tradeoffs between functionality, chemical stability, and biocompatibility. Other constraints are related to the chemical properties of the products—polyphenols exhibit poor membrane permeability, rapid metabolism, and poor bioavailability and UV degradation, despite free radical grafting [73], step growth, free radical, and enzyme-catalyzed reactions. A vast majority of research in antioxidant materials so far has been focused on the entrapment or encapsulation of low-molecular-weight and enzymatic antioxidant compounds in polymersomes, micelles, or nanoparticles that often exhibit poor long-term stability. The structural incorporation of antioxidant moieties in polymers or direct polymerization while maintaining their intrinsic antioxidant properties has been attracting increasing interest from the research community. Polymerized antioxidants can be produced through grafting or backbone incorporation, and offer the potential advantages of protecting the antioxidant from inactivation, delivering continuous antioxidant protection as long as the polymer exists, as well as a relatively high mass of antioxidant payload.

Potential industrial applications of antioxidants in the food production industry are diverse, especially in the forms of blends with bio-polyether (PEO), bio-polyester (PLA), and commercial starch-based polymer (Mater-Bi) for photo-stabilization. The blended products exhibit better tensile strength, photostability, and elongation at break and modulus of elasticity. Active polymer packaging is becoming increasingly important as an emerging technology [112] that can significantly improve the quality and stability of food, eliminating direct addition of chemicals and the need to make significant changes in production processes. If antioxidant polymers are brought into packaging systems, major improvements in maintaining the stability of oxidation-sensitive food products can be made. Antioxidant polymers have been proven to be scavengers that function by reducing the presence of reactive oxygen species, which act as initiators of oxidation processes. In this review, we have seen that antioxidant polymers can be specifically designed and optimized for each specific product or application and in brief, they can be implemented by the food industry as new emerging sustainable polymeric materials.

## Figures and Tables

**Table 1:** Active compounds, natural sources of antioxidants, and their use in different food matrices [4].

Natural Source	Main Active Compound	Food Matrix
Fennel and chamomile aqueous extracts	Phenolic compounds	Biscuits
		Cottage cheese
		Yogurt
Olive leaf and cakes extracts byproducts	Phenolic compounds	Antioxidant film
Litchi fruit pericarp extract	Phenolic compounds	Cooked nuggets
Green tea extract	Polyphenols	Sunflower oil
Cloves and cinnamon	Phenolic compounds	Meat samples
Tomato pomace extract	Carotenoids	Lamb steaks packaged
*Ginkgo biloba* leaves extract	Polyphenols	Pork meat
Cloudberry, beetroot, and willow herb	Flavonoid	Cooked pork patties
Canola olive oils, rice bran, and walnut	Polyphenols, vitamins E and B	Pork frankfurters
Coffee	Chlorophylls and carotenoids	Not defined
Wine	Phenolic compounds	Meat, fish, cereals

**Table 2:** Extraction techniques for different classes of naturally occurring antioxidants [4].

Extraction Process	Source	Antioxidant Extracted
Organic Solvents:		
Ethanol, dichloromethane, hexane	Coffee leaves	Chlorophylls and carotenoids
Ethanol, acetone, and water	*Baccharides* species	Phenolic content
	Sweet potato	Polyphenols and anthocyanins
Methanol, ethanol, and acetone	Spent grain	Phenolic content
	Spice herb	Phenolic content
	Peel of eggplant	Total phenolics, flavonoids, tannins, and anthocyanins
Supercritical fluid extraction (SFE)	Mango peel	Carotenoids
	Apple pomace	Phenolic compounds
	Myrtle leaves and berries	Phenolic acids, flavonoids, and anthocyanins
	Green algae	Carotenoids and phenolic compounds
	Cape gooseberry	Phenolic compounds and β-carotene
High Hydrostatic Pressure (HHP)	Red macroalgae	Proteins, polyphenols, and polysaccharides
	Tomato pulp	Flavonoids and lycopene
	Watercress	Phenolic acids and flavonoids from watercress
	Papaya seeds	Phenolic content
Pressurized liquid extraction (PLE)	Peppermint	Phenolic compounds and essential oils
	Carrot byproducts	Carotenoids
Ultrasound-assisted extraction	Green propolis	Phenolic compounds
Microwave-assisted extraction (MAE)	Pomegranate peels	Phenolic compounds
	*Phalera macrocarpa* fruit peel	Phenolic compounds

**Table 3:** Cost of recovering antioxidants from selected agro-wastes and the phenol content [24].

Agro-Waste	Annual	Cost	Calculated Total Cost	Antioxidant	Total Phenol
	production	($/kg)			content
	(thousand tons)				(mg GA/g)
Red Grape	100	0.050	5	αtocopherol,	~0.02
				Gallic acid	
Turmeric				Curcumin	~3.1
Coffee grounds	0.005	0.050	0.00025	Melanoidin,	17
				Chalcogenic	
				acid	
Orange peel	20	0.050	1	Ascorbic acid	~1.3
				and Flavonoids	
Irganox I10	Not known	6.36		Four substituted	
				2,6 di-tertiary	
				butyl phenols	

**Table 4:** Function of selected genes [16].

Genes	Plant Species	Functions
*4CL*	*Oryza sativa* (*OsAAE3*)	Decreased lignin accumulation and increased sensitivity to rice blast
*CCR*	*Paspalum dilatatum* (*PdCCR1*)	Increased lignin content and altered lignin deposition
*CAD*	*Populus trichocarpa* (*PtrCAD1*)	Modified lignin content and structure
*MOMT*	*Arabidopsis thaliana* (*MOMT4*)	Depressed lignin biosynthesis and increased saccharification yields
*COMT*	*Sorghum bicolor* (*SbCOMT*)	Methylate the tricin precursors and participated in S lignin biosynthesis

**Table 5:** Second-order kinetics of lignin-based materials [7].

Sample	*p* (30 min)	CV (%)	Linear Fit	The Domain of Validity (pNCO)	[NCO]0	Kapp 10^3^ (L/mol/s)
(PCL400/1/0)	0.93	0.41	1 + 0.525t	0.93	4.72	1.87
A(PCL400/1/10)	0.91	0.19	1 + 0.491t	0.87	3.52	2.32
A(PCL400/1/15)	0.87	0.67	1 + 0.461t	0.82	3.54	2.16
A(PCL400/1/20)	0.84	0.43	1 + 0.469t	0.71	3.57	2.20
A(PCL400/1/25)	0.77	0.94	1 + 0.377t	0.66	3.59	1.76
A(PCL750/1/15)	0.84	1.30	1 + 0.350t	0.73	2.47	2.36
A(PCL750/1/20)	0.79	1.33	1 + 0.323t	0.66	2.56	2.08
A(PCL750/1/25)	0.74	1.18	1 + 0.327t	0.56	2.66	2.07
(PCL1000/1/0)	0.79	1.12	1 + 0.123t	0.79	1.73	1.16
A(PCL1000/1/10)	0.82	0.40	1 + 0.212t	0.75	1.98	1.77
A(PCL1000/1/15)	0.78	0.42	1 + 0.247t	0.63	2.11	1.98
A(PCL1000/1/20)	0.75	1.04	1 + 0.260t	0.57	2.23	1.94
A(PCL1000/1/25)	0.70	1.55	1 + 0.257t	0.48	2.35	1.84

**Table 6:** Yields, MW, and PD from acrylamide and styrene polymerization as initiators [76].

**Initiator: Styrene Polymerization**		
Yield %	MW [×10^−3^ g·mol^−1^]	PD
17	27	2.1
59	68	2.0
14	80	2.0
14	97	2.2
14	57	1.6
**Initiator: Acrylamide Polymerization**		
Yield %	MW [×10^−3^ g·mol^−1^]	PD
93	124	2.5
84	56	2.9
76	5	4.4
72	27	3.3
78	85	2.7
38	10.5	3.9

**Table 7:** Changes in the mechanical properties (elastic modulus, tensile strength, and elongation at break) before and after stabilization with quercetin additives (Q): Cyasorb and synthetic Light Stabilizers (LS) [78].

Samples	E, MPa		TS, MPa	EB,%	
PLA	1556	±52	47.1 ± 2.5	9.3 ± 1.5	
PLA/Q	1684	±56	44.8 ± 2.2	14.8	±2.0
PLA/LS	1843	±62	51.2 ± 2.5	19.5	±2.5
MB	105	±5	24.2 ± 1.2	435	±22
MB/Q	119	±6	27.0 ± 1.3	470	±24
MB/LS	120	±6	24.6 ± 1.2	440	±22
PEO	60.5 ± 2.3		3.5 ± 0.3	105 ± 5.0	
PEO/Q	55.2 ± 2.5		3.1 ± 0.2	110 ± 6.0	
PEO/LS	54.2 ± 2.3		3.2 ± 0.3	115 ± 7.5	

**Table 8:** Classifications of terpenes and their potential application as pesticides [91].

Terpene Type	Function	Features
Limonene	Limonene enhances the properties of other terpenes.	Redistilled limonene has less odor, more stable than d-limonene
Beta-ionone	Antibacterial and antifungal properties	Beta-ionone has prophylactic value.
Geraniol	Like beta-ionone, antibacterial and antifungal properties.	Geraniol gives a pleasant fragrance.
Eugenol	Clove oil; anesthetic (anti-itch), antibacterial antifungal properties.	Contain a distinct fragrance which is like geraniol
Myrcene	Possesses antifungal, antibacterial properties	Famous for its fragrance properties

**Table 9:** Mediated copolymerization of PO, time conversion, MW and Tg [88].

Entry Monomer	Cat.	[PO]/[2][b]	[ah-1 c]/[cat.-2][b]	Time	Conversion[c]	Mn[d]	Mw/Mn[d]	cis[e]	Tg[f]
				[h]	[%]	[kDa]		[%]	[8C]
1	cat.-2 a	500	100	0.75	86	11.4	1.25	>99	105
2	cat.-2 a	500	100	2.0	>99	18.8	1.66	52	91
3[g]	cat.-2 a	100	150	3.0	>99[h]	12.2	1.22	>99	102
4	cat.-2 b	500	100	2.0	76	9.0	1.18	>99	101
5	cat.-2 b	500	100	3.0	>99	14.6	1.72	10	78
6	cat.-2 c	500	100	1.0	63	7.4	1.11	>99	97
7	cat.-2 c	500	100	2.5	>99	10.2	1.16	97	103
8	cat.-2 c	500	100	5.0	>99	9.8	1.16	95	102
9	cat.-2 c	1000	200	4.0	>99	22.5	1.28	88	104
10	cat.-2 c	2000	400	9.0	>99	36.7	1.30	94	107
11	cat.-2 c	4000	800	20.5	>99	55.4	1.29	98	109

**Table 10:** Microbial-based synthetic routes for polylimonene through microbial mediated synthesis processes [93].

Host	Engineering Design	Limonene Synthase Origin, (+/−)-Limonene, Accession Number
*E. coli* BLR (DE3) codon +	Abies grandis tGPPS	*Mentha spicata*, (−), L13459
*E. coli* DH1 ∆acrAB	HMGS and tHMGR from Staphylococcus aureus Codon optimized MVK, PMK, and PMD from *S. cerevisiae* AACT and IDI from *E. coli* tGPPS from Abies grandis One plasmid containing the mevalonate pathway genes and one plasmid with tGPPS and tLS A. Borkumensis efflux pump	*M. spicata*, (−), L13459, codon-optimized
*E. coli* DH1	HMGS and tHMGR from Staphylococcus aureus Codon optimized MVK, PMK, and PMD from *S. cerevisiae* AACT and IDI from *E. coli* tGPPS from Abies grandis One plasmid	*M. Spicata*, (−), accession number not clear, codon-optimized
*E. coli* BL21(DE3)	HMGS and tHMGR, MVK, PMK, and PMD from *S. cerevisiae* AACT and IDI from *E. coli* tGPPS from Abies grandis/GPPS from Streptomyces sp. strain KO-3988	*M. Spicata*, (−), L13459, codon-optimized
*Synechocystis* sp. PCC 6803	DXS, IDI, and CrtE from Synechocystis	*Schizonepeta tenuifolia,* enantio-selectivity not clear, AF282875
*Synechococcus* sp. PCC 7002	Wild-type and ΔglgC background were compared	*M. Spicata*, (−), Q40322, codon-optimized
*S. cerevisiae* AE9	Yeast FPPS (ERG20 K197G) mutated to partly produce GPP	*Perilla frutescens*, (−), KM015220 and Citrus limon, (+), AF514287
*S. cerevisiae* EPY210C	tHMGR from *S. cerevisiae* upc2–1 transcription factor	*M. spicata*, (−), L13459 and C. Limon, (+), AF514287, codon-optimized

**Table 11:** Antioxidant migration under microwaving and heating conditions.

Microwaving			Heating	
Sample	I168	I1010	I168	I1010
90/10 iso-octane/ethanol, 80 °C				
PPC	509	502	686 ± 31 a	726 ± 64 a
PPR	4735	1283	4955 ± 204 a	1342 ± 217 a
PP	615	190	748 ± 85 a	277 ± 45 a
Iso-octane, 80 °C				
PPC	–	–	777	765
PPR	–	–	4909	1477
PP	–	–	780	429
3% acetic acid, 100 °C				
PPC	b	9	b	b
3% acetic acid, 120 °C				
PPC	b	9	b	b
10% ethanol, 120 °C				
PPC	b	6	b	b

## Data Availability

The data presented in this study are available on request from the corresponding author.
